# Supplementary material for: Renaissance Distribution for Statistically Failed Experiments
Source: Int J Mol Sci. 2019 Jul 2;20(13):3250. doi: 10.3390/ijms20133250 (PMC6651062; doi:10.3390/ijms20133250)
Supplement: Supplementary file 1 [file ijms-20-03250-s001.pdf]

International Journal of *Molecular Sciences*

Supplementary Materials

Article

Renaissance Distribution for Statistically Failed Experiments

Roman Popov, Girish Karadka Shankara, Clemens von Bojničić-Kninski and Alexander Nesterov-Mueller *

Institute of Microstructure Technology, Karlsruhe Institute of Technology (KIT), 76344 Eggenstein-Leopoldshafen, Germany; roman.popov@kit.edu (R.P.); girish.shankara@kit.edu (G.K.S.); clemens.bojnicic-kninski@kit.edu (C.B.-K.);

* Correspondence: Alexander.Nesterov-Mueller@kit.edu (A.N.-M.)

An example of Renaissance distribution with EXCEL

1. Open an a EXCEL sheet. Copy your data into a column;
2. Generate the graph (Fig. S1, the signals from 100 identical experiments have been taken);
3. Calculate the mean value M and its standard deviation STDV (For the data in Figure S1: M = 173.7; STDV = 106.2).
   The experiment has failed statistically because STDV is very high.
4. Apply to the column the command "Sort and Filter" and "from largest to smallest". The graph gets the form presented in Figure S2.

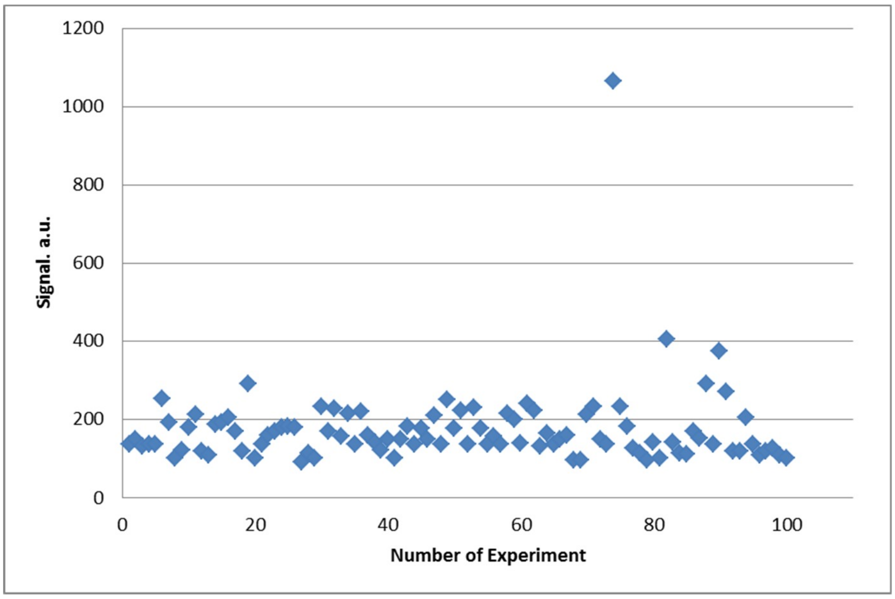

Figure S1: The distribution of the signal over 100 identical experiments.

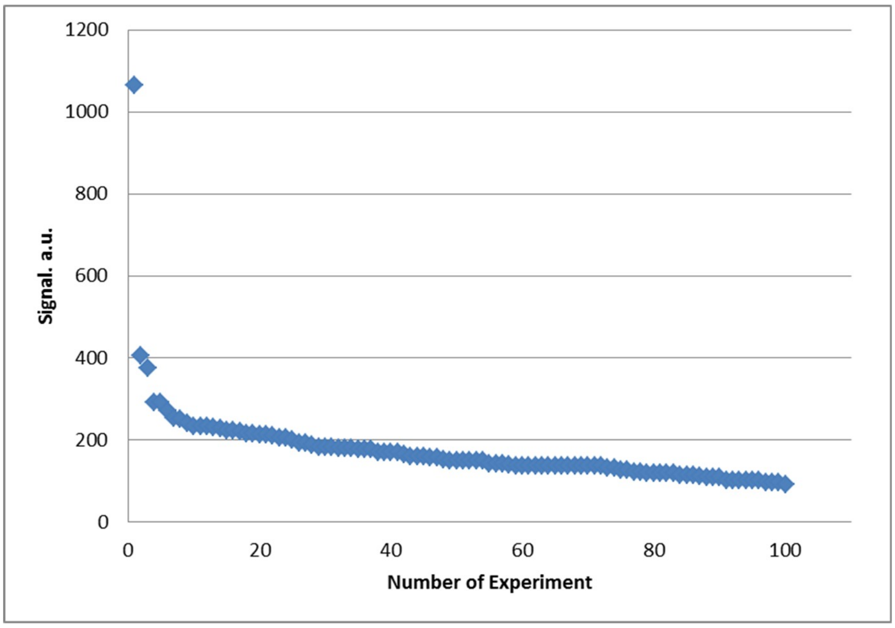

Figure S2: The distribution of the signal over 100 identical experiments after sorting the signal from Figure 2 “from largest to smallest”.

5. Open the menu with the right mouse click on the dotted line and choose the button “Insert trend line”. A window “Trend line options” appears. Choose “logarithmic “ and the last two options “Show the formula in diagram” and “Show the approximation rate in diagram”. After performing these options, Renaissance distribution is generated (Figure S3, black line).

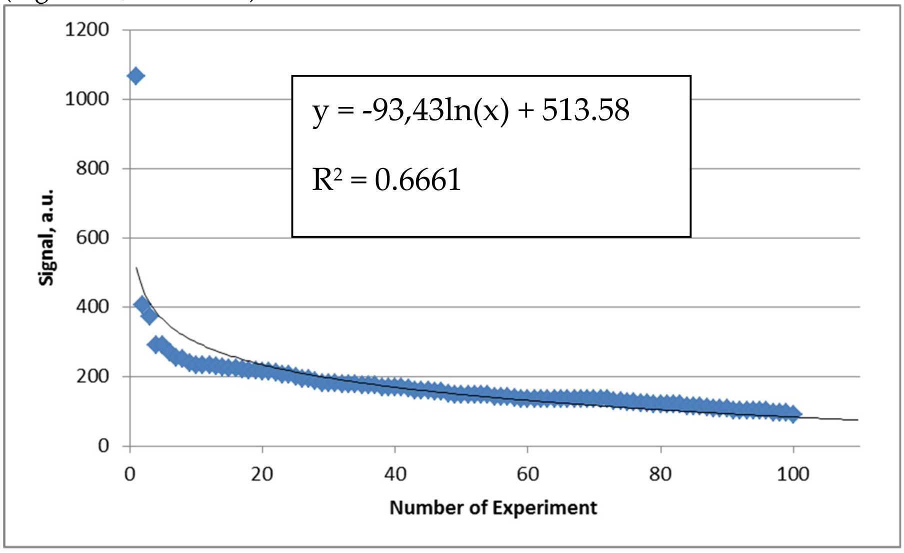

Figure S3: The distribution of the signal over 100 identical experiments and corresponding Renaissance distribution (black line) with $R^2 = 0.66$. ($R^2 \in (0:1)$).

6. Improve Renaissance approximation – R^2 by deleting critical points. One point S = 1065 is identified as artifact. Its deletion leads to significantly better approximation (Figure S4): $R^2 = 0.9719$.

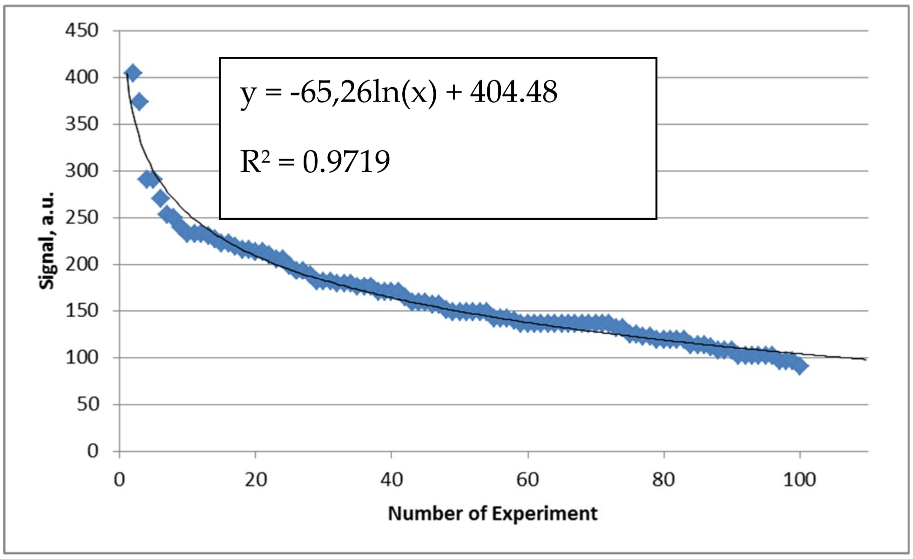

Figure S4: The distribution of the signal over 99 identical experiments and corresponding Renaissance distribution (black line) with $R^2 = 0.9719$.

7. Thus, the truth value is 404.48 with a approximation precision $R^2 \approx 0.97$. In contrast, the mean value of the original signals is 173.7 with standard deviation of 106.2. While the deletion of the point S = 1065 (artifact) from the original data is justified by reaching higher R^2 in the case of Renaissance distribution, its deletion would be a voluntary action in the case of the classical statistics based on the calculation of mean value and its deviations.

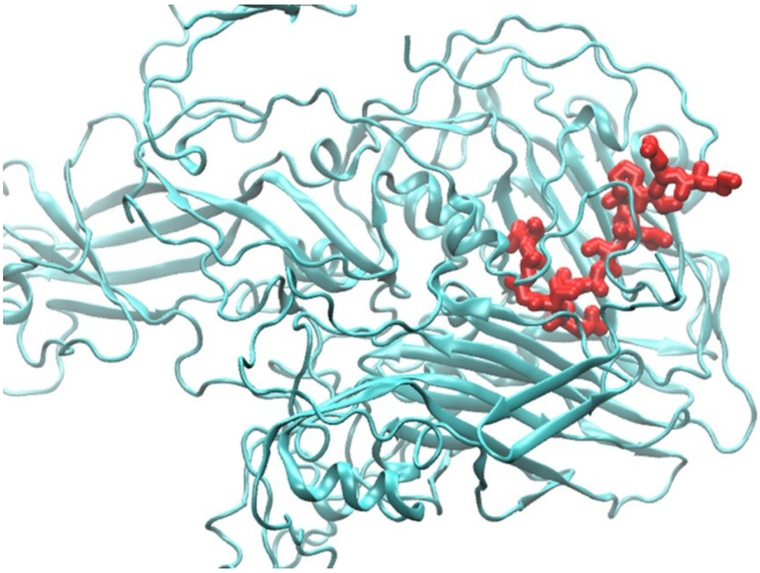

Figure S5: The location of the target peptide in the capsid protein VP1 of the poliovirus. The location was obtained using VMD: Visual Molecular Dynamics [10]. The 3D structure of the capsid protein VP1 of the poliovirus is available online in the Protein Data Bank [11].
